# Purulent pericarditis caused by *Nocardia*: a case report and literature review

**DOI:** 10.3389/fcvm.2025.1465047

**Published:** 2025-09-05

**Authors:** Xinxin Zhong, Ao Lin, Fu Cao, Shihao Jiang, Yuying Ruan, Shuting Li, Li Zhong, Zhiyi He, Jian Luo

**Affiliations:** ^1^ Department of Pulmonary and Critical Care Medicine, Red Cross Hospital of Yulin City, Yulin, Guangxi, China; ^2^ Department of Cardiothoracic Surgery, Red Cross Hospital of Yulin City, Yulin, Guangxi, China; ^3^ Department of Laboratory Medicine, Red Cross Hospital of Yulin City, Yulin, Guangxi, China; ^4^ Department of Pulmonary and Critical Care Medicine, The First Affiliated Hospital of Guangxi Medical University, Nanning, Guangxi, China

**Keywords:** *Nocardia*, pericarditis, antibiotic therapy, pericardiocentesis, AIDS

## Abstract

Purulent pericarditis caused by *Nocardia*, a rare opportunistic infection associated with a high mortality, is frequently misdiagnosed as *Mycobacterium tuberculosis* (MTB) or other bacterial infections. We report a case of *Nocardia*-induced purulent pericarditis in a patient with acquired immune deficiency syndrome (AIDS). The patient experienced multiple misdiagnoses and received inappropriate anti-tuberculosis therapy. Timely pericardial puncture and subsequent culture of the pericardial effusion identified *Nocardia*, prompting initiation of appropriate antibiotic therapy which led to clinical cure. This case report underscores the importance of broad differential diagnostic considerations in purulent pericarditis and emphasizes that prompt initiation of *Nocardia*-directed antibiotic therapy, guided by microbiological identification, is crucial for timely diagnosis and management. Additionally, we review and summarize previously reported cases of laboratory-confirmed *Nocardia* pericarditis in AIDS patients.

## Background

*Nocardia* species are filamentous, partially acid-fast, aerobic, Gram-positive bacteria ubiquitous in soil and water (1). The skin, lungs, and brain are the most common sites of infection; however, disseminated disease can potentially affect any organ (2–7). *Nocardia* can infect a small minority of immunocompetent individuals (8). *Nocardia* is primarily an opportunistic pathogen, with most cases reported in patients with acquired immune deficiency syndrome (AIDS) or other conditions impairing cell-mediated immunity, such as hematologic malignancies (leukemia, lymphoma), solid organ transplantation, and prolonged glucocorticoid therapy (4, 9, 10). Consequently, mortality is significantly influenced by these underlying risk factors (11).

*Nocardia* pericarditis is a rare opportunistic infection characterized by high mortality and nonspecific symptoms. Due to similarities in staining characteristics (partially acid-fast) and morphology with *Mycobacterium tuberculosis* (MTB), nocardiosis is frequently misdiagnosed as tuberculosis (TB) (12). This report describes an AIDS patient who developed *Nocardia* pericarditis.Despite experiencing multiple misdiagnoses and receiving inappropriate anti-tuberculosis therapy, timely pericardial puncture and effusion culture ultimately identified *Nocardia*. This led to the initiation of appropriate antibiotic therapy and subsequent clinical cure. We aim to emphasize the critical importance of considering *Nocardia* in the differential diagnosis of purulent pericarditis and the need for timely, targeted antibiotic therapy based on microbiological identification to facilitate effective management of *Nocardia* pericarditis. Additionally, we discuss previously reported cases of *Nocardia* pericarditis in AIDS patients.

## Case presentation

A 52-year-old Chinese male presented with a chief complaint of fever, fatigue, and dyspnea persisting for two weeks. Based on the patient's immunocompromised status (recently diagnosed AIDS), exudative pericardial effusion, pericardial fluid adenosine deaminase (ADA) level >45 U/L [a biomarker with 98% positive predictive value for TB pericarditis in TB-endemic areas (13)], elevated erythrocyte sedimentation rate (ESR), and positive MTB IgG serology (indicating prior TB exposure), he was initially diagnosed with tuberculous pericarditis (TBP). This diagnosis was made despite a negative IGRA result, which was interpreted in light of the known limitation of IGRA sensitivity in HIV-infected individuals (14).

**Figure 1:**
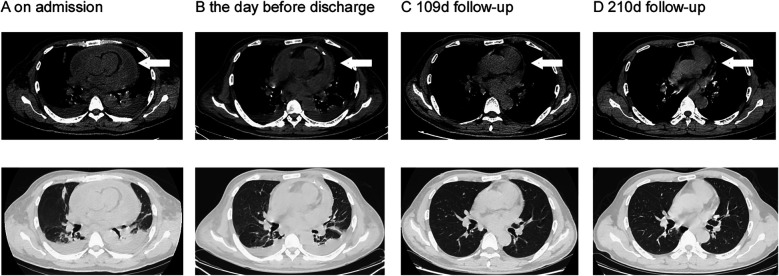
Serial imaging changes during hospitalization and follow-up. The upper section displays the mediastinal window, while the lower section represents the lung window. The white arrow points to the effusion of pericardial. **(A)** Shows the chest CT scan obtained on admission, revealing pericardial effusion and symmetrical pleural effusion with localized atelectasis in the lower lobes of both lungs. **(B)** Shows Chest CT at discharge demonstrating significant resolution of pericardial effusion with small residual fluid. **(C,D)** Shows follow-up chest CT scans at the outpatient clinic, demonstrating complete absorption of pericardial effusion and symmetrical pleural effusion.

The patient underwent pericardial puncture with indwelling catheter placement for continuous drainage and received one week of anti-tuberculosis therapy for presumed TBP at a local hospital. However, his symptoms failed to improve, prompting transfer to our hospital for further management. He had a recent diagnosis of AIDS, with no prior CD4+ T-cell count monitoring or antiretroviral therapy (ART). No significant comorbidities, smoking history, or known drug allergies were reported. Vital signs on admission were: temperature 36.7 °C (post-antipyretic), blood pressure 109/84 mmHg, heart rate 107 beats/min, respiratory rate 22 breaths/min, and oxygen saturation 96% with supplemental oxygen at 2 L/min. Physical examination revealed bilateral diminished breath sounds, enlarged cardiac dullness (suggesting significant pericardial effusion), and bilateral pitting edema of the lower extremities. Purulent pericardial fluid drained continuously via the indwelling catheter. Laboratory results demonstrated a markedly reduced peripheral blood lymphocyte percentage of 3.3% (reference range: 20%–40%) alongside significantly elevated inflammatory markers: C-reactive protein (CRP) 146.34 mg/L (reference: <8.2 mg/L) and procalcitonin (PCT) 1.05 ng/ml (reference: <0.05 ng/ml). The IGRA returned negative. Peripheral blood lymphocyte subset analysis revealed profound depletion of CD3+ CD4+ T-cells (0.01%; reference range: 27%–51%) and complete inversion of the CD4+/CD8+ T-cell ratio (0.00), consistent with WHO Stage 3 HIV/AIDS (15). Peripheral blood lymphocyte examination revealed increased peripheral blood B-cell percentage (46.58%; reference range: 5%–18%), indicative of HIV-associated polyclonal B-cell dysregulation. Serological testing was positive for MTB IgG antibody (TB-IgG), suggesting prior tuberculosis exposure. Chest CT scans revealed bilateral symmetric pleural effusions with localized atelectasis in the lower lobes of both lungs, and massive pericardial effusion (Figure 1A). Echocardiography confirmed a large pericardial effusion. Given the absence of neurological symptoms, cranial imaging was not performed.

**Figure 2:**
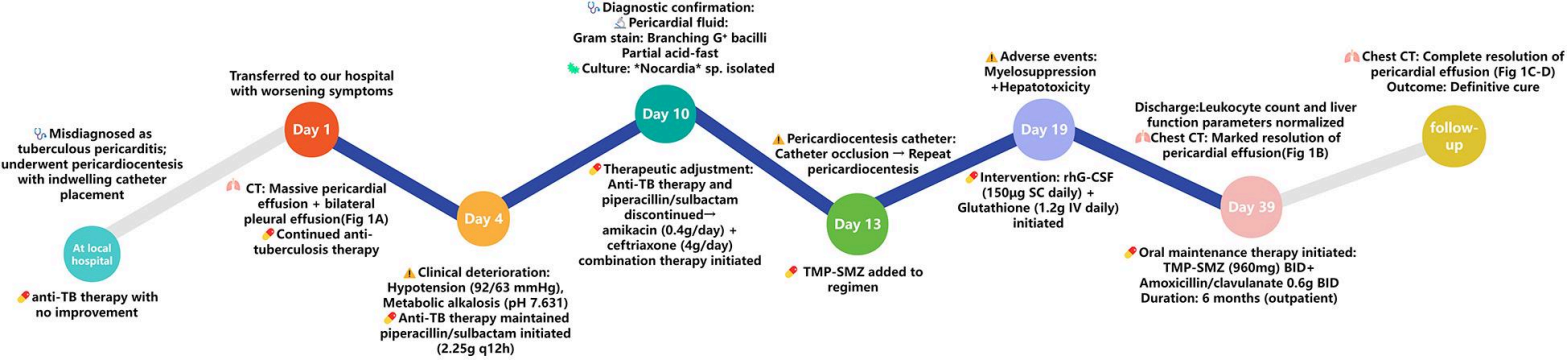
Clinical timeline of *Nocardia* purulent pericarditis in an AIDS patient.

Based on the initial assessment from the local hospital and subsequent diagnostic findings upon admission, the patient continued to receive anti-tuberculosis therapy. Despite this treatment, the patient's condition deteriorated, with progressive worsening of symptoms, hypotension (blood pressure 92/63 mmHg), and increased shortness of breath. Persistent large-volume drainage of viscous, purulent pericardial fluid via the indwelling catheter was noted. On the fourth day of hospitalization, laboratory findings revealed a severe acid-base disturbance (arterial blood pH 7.631), hypokalemia (serum potassium 2.90 mmol/L), and hypochloremia (serum chloride 83 mmol/L), culminating in metabolic alkalosis. The pathogenesis of these electrolyte imbalances was thought to be related to electrolyte losses associated with an underlying infection. Therapeutic interventions included aggressive intravenous repletion with potassium chloride and aggressive ventilation. Subsequent arterial blood gas analysis demonstrated that both pH and electrolyte levels were approaching normal. Given the absence of fibrotic changes on chest imaging, the differential diagnosis focused on inflammatory pericardial diseases causing pericardial effusion. The following conditions were considered and required exclusion: (1) Non-tuberculous mycobacterial (NTM) infection: This was considered less likely due to the absence of characteristic pulmonary lesions often associated with disseminated NTM disease in immunocompromised hosts. (2) TBP: This diagnosis became increasingly unlikely given the persistence of the patient's symptoms and purulent drainage despite ongoing anti-tuberculosis therapy. (3) Fungal pericarditis: While the negative serum 1,3-β-D-glucan (BDG) test argued against some fungal etiologies, it does not definitively exclude fungal infection. Empiric intravenous therapy with piperacillin/sulbactam (2.25 g every 12 h) was initiated due to disease progression and elevated inflammatory markers.

Fungal and routine bacterial cultures of blood and pericardial fluid, viral molecular testing, and pericardial fluid Xpert MTB/RIF assay (which excluded *M. tuberculosis*) were all negative. However, microscopic examination of the pericardial fluid revealed branching Gram-positive bacilli on Gram stain, which exhibited partial acid-fastness on modified Ziehl-Neelsen stain, suggesting possible *Nocardia* infection. The pericardial fluid was subjected to Gram staining (revealing branching Gram-positive bacilli), modified Ziehl–Neelsen staining (confirming partial acid-fastness), and prolonged aerobic culture on sheep blood agar at 37 °C for 10 days. This extended incubation period is critical to avoid false negatives in immunocompromised hosts (16). Growth characteristics (dry, chalk-white colonies observed by day 7) and microscopic morphology confirmed the isolate as *Nocardia* genus. Species-level identification was not performed due to lack of access to MALDI-TOF mass spectrometry or 16S rRNA sequencing. Due to resource limitations that prevented species identification, therapy could not be optimized based on specific species susceptibility patterns. Literature indicates variable sulfonamide susceptibility among common pathogenic species like *N. asteroides* (17–19) complex and *N. brasiliensis* (20). Therefore, broad-spectrum combination therapy was necessitated. Susceptibility testing demonstrated susceptibility to amikacin, amoxicillin/clavulanic acid, ceftriaxone, imipenem, ciprofloxacin, minocycline, and trimethoprim-sulfamethoxazole (TMP-SMZ). Antitubercular medications and piperacillin/sulbactam were discontinued, and combination therapy with intravenous ceftriaxone (4 g/day) plus intravenous amikacin (0.4 g/day) was initiated.

On hospital day 13, persistent drainage of thick, purulent exudate led to complete occlusion of the pericardiocentesis catheter. A repeat pericardiocentesis was performed with placement of a new drainage catheter. TMP-SMZ (trimethoprim 160 mg/sulfamethoxazole 800 mg twice daily) was added to the ongoing empirical antibacterial regimen of amikacin (0.4 g/day) and ceftriaxone (4 g/day). Subsequently, the patient demonstrated gradual symptomatic improvement accompanied by reduced pericardial fluid output.

However, drug-induced myelosuppression and hepatotoxicity developed during antimicrobial therapy, manifested as significant leukopenia (white blood cell nadir: 1.27 × 10^9^/L), anemia (hemoglobin nadir: 61 g/L), and elevated transaminases. These adverse events were causally linked to high-dose ceftriaxone administration (4 g/day) and TMP-SMZ, consistent with the documented risks of myelosuppression and hepatotoxicity associated with this agent (21–24). Following subcutaneous recombinant human granulocyte colony-stimulating factor (rhG-CSF; 150 μg once daily) combined with intravenous glutathione (1.2 g daily) for hepatoprotection, leukocyte counts returned to normal range (6.39 × 10^9^/L) and transaminase levels declined below the upper limit of normal (<40 U/L).

A chest CT scan prior to discharge showed significant resolution of the bilateral pleural and pericardial effusions (Figure 1B). Corresponding echocardiography indicated a small residual pericardial effusion. At outpatient follow-up, the patient received oral TMP-SMZ [trimethoprim 160 mg/sulfamethoxazole 800 mg (0.96 g) twice daily] combined with oral amoxicillin/clavulanic acid [0.686 g (e.g., amoxicillin 600 mg/clavulanic acid 86 mg) twice daily] for an additional six months. Subsequent follow-up visits revealed no recurrence of infectious symptoms and no severe adverse reactions to the antimicrobial regimen. Repeat chest CT scan and echocardiography showed complete resolution of the pericardial effusion with no evidence of residual fibrosis or calcification (Figures 1C,D). The clinical timeline of the patient's diagnosis, treatment, and adverse events is summarized in Figure 2.

## Discussion and conclusions

While nocardiosis is uncommon in AIDS [incidence: 0.2%–1.8% (25)], pericardial involvement is exceptionally rare. Our literature review (Table 1) reveals key trends: 81.8% male predominance (9/11 cases), 54.5% incidence of cardiac tamponade (6/11), and 27.3% mortality (3/11)—underscoring the virulence of this pathogen in immunocompromised hosts.

**Table 1:** Summary of all published cases of nocardial pericarditis in AIDS.

Age	Sex	cardiac tamponade	CD4 cell count (cells/mm3)	Misdiagnose tuberculous pericarditis	Drainage/surgery/drainage flow	Drug treatment	Antiretroviral therapy	Outcome	References
32	Male	No	48	No	Subxiphoid pericardiostomy (1,100 ml fluid)	SMX/TMP, IV	Not reported	Recovered	Holtzet al. (6)
34	Male	No	66	No	Pericardiostomy (400 ml fluid)	SMX/TMP, IV, 3 weeks	Not reported	Died	Holtzet al. (6)
34	Male	Yes	239	No	Pericardiostomy (400 ml fluid)	SMX/TMP, IV, 5 days → Sulfadiazine, PO, 3 months	Yes	Recovered	Rivero et al. (21)
42	Male	No	91	Yes	Pericardial window	SMX/TMP but allergy → Ceftriaxone + Minocycline, 6 weeks	Not reported	Recovered	Ramanathan and Rahimi (16)
24	Female	Yes	Not reported	No	Pericardial aspirations with indwelling catheter (900 ml fluid)	SMX/TMP, PO	Not reported	Recovered	Leang et al. (7)
44	Male	No	42	Yes	Pericardiocentesis followed by surgical drainage with pericardial window	SMX/TMP, IV → PO	No	Recovered	Jinno et al. (18)
35	Female	Yes	32	Yes	Pericardiocentesis (500 ml fluid)	SMX/TMP + ceftriaxone + doxycycline, IV → SMX/TMP + doxycycline, PO	Yes	Recovered	Chandrashekar et al. (25)
37	Male	Yes	50	No	Subxiphoid pericardial drain (800 ml fluid)	Imipenem/cilastatin + SMX/TMP, IV → SMX/TMP, PO	No	Recovered	Aisenberg and Martin (19)
32	Male	Yes	17	Yes	Emergency pericardiocentesis	Imipenem/cilastatin + SMX/TMP, IV	Yes	Died	Laksananun et al. (53)
32	Male	Yes	4	No	Pericardiocentesis	→SMX/TMP + Imipenem/cilastatin, IV, Continue anti-TB therapy (HREZ)	Not reported	Died	Griessel et al. (27)
52	Male	No	Not reported	No	pericardiocentesis with indwelling catheter (4,280 ml of pus)	SMZ/TMP, PO + ceftriaxone + Amikacin, IV → SMZ/TMP and amoxicillin/clavulanic acid, PO	No	Recovered	Xinxin Z et al. (current study)

AIDS, acquired immune deficiency syndrome; SMX/TMP, sulfamethoxazole/trimethoprim; IV, intravenous; PO, per oral; TB, tuberculosis; H, isoniazid; R, rifampin, Z, pyrazinamide; E, ethambutol.

The clinical presentation of *Nocardia* pericarditis is nonspecific. Common symptoms, as reported in the literature, include fever, night sweats, dyspnea, anorexia, weight loss, chest pain, chills, cough, and fatigue (26, 27). Prior to microbiological confirmation, the clinical diagnosis of *Nocardia* pericarditis is challenging and requires differentiation from other causes of purulent pericarditis (Table 2).

**Table 2:** Comparative characteristics of different etiologies of purulent pericarditis.

Characteristics	Nocardial pericarditis (16, 53)	Tuberculous pericarditis (32)	MRSA pericarditis (34, 36)	Viral pericarditis (Influenza—related) (40)	Fungal pericarditis (41, 44)
Susceptible Populations	Immunocompromised individuals (such as those with AIDS)	People in developing countries, HIV—infected individuals	Long—term hospitalized patients, those with chronic underlying diseases	All age groups, more common in men and the elderly	Severely immunosuppressed patients
Typical Symptoms	Fever, dyspnea, purulent pericardial effusion	Low—grade fever, night sweats, elevated adenosine deaminase	High fever, sepsis, cardiac tamponade	Chest pain, influenza—like symptoms, tachycardia	Fever, weight loss, elevated BDG
Gold Standard for Diagnosis	Pericardial effusion culture (should be extended to at least 10 days)	Xpert MTB/RIF	MRSA culture from pericardial effusion	Viral nucleic acid detection	Fungal culture/BDG detection
First—line Treatment	SMZ/TMP + ceftriaxone/amikacin	Combined anti—tuberculosis drug treatment	Vancomycin	Supportive treatment + antiviral drugs	Amphotericin B/echinocandins
Mortality Rate	28.3% (in AIDS—complicated cases)	High (when not treated in a timely manner)	20.5% (in adults)	Low (with timely treatment)	High (in immunosuppressed patients)

AIDS, acquired immune deficiency syndrome; SMX/TMP, sulfamethoxazole/trimethoprim; BDG, β-D-glucan; MRSA, methicillin-resistant Staphylococcus aureus.

In developing countries, TBP remains the predominant cause of purulent pericarditis (28). *Mycobacterium tuberculosis* can disseminate to the pericardium via retrograde lymphatic spread, hematogenous dissemination, or direct extension from adjacent lung, pleural, or spinal foci (29). In patients co-infected with HIV, pericardial effusion cultures are positive for MTB in only approximately 35% of TBP cases (30). Xpert MTB/RIF and Xpert MTB/RIF Ultra are automated, cartridge-based nucleic acid amplification tests (NAATs) capable of detecting MTB and simultaneously assessing rifampicin resistance within two hours (31). In a study evaluating Xpert MTB/RIF accuracy for TBP and other forms of extrapulmonary TB, sensitivity was 63.8% with high specificity (100%) (32). Furthermore, in high TB prevalence areas, IGRAs have limited utility for diagnosing active TB, as positive results only indicate prior MTB antigen exposure and do not reliably distinguish latent from active infection (33). In the present case, the patient was initially misdiagnosed with TBP. Therefore, if patients fail to respond to appropriate anti-tuberculosis therapy, clinicians should consider the possibility of *Nocardia* infection and pursue microbiological identification and susceptibility testing promptly. In this case, the negative pericardial fluid Xpert MTB/RIF, cultures, and IGRA, combined with the lack of response to anti-tuberculosis therapy, definitively excluded TBP.

*Methicillin-resistant Staphylococcus aureus* (MRSA) pericarditis and pericardial abscesses are rare and typically associated with chronic comorbidities or prolonged healthcare exposure (34, 35). Pericardial effusion occurs in 94.9% of MRSA pericarditis cases, with cardiac tamponade developing in 83.8% (36). In reported cases, MRSA is invariably isolated from pericardial fluid, and bacteremia is present in 64.1% (36). The adult mortality rate is 20.5%, with median survival of 21.8 days, primarily due to multi-organ dysfunction from septic shock (36). In this case, the patient lacked significant healthcare exposure or chronic comorbidities predisposing to MRSA, and both blood and pericardial fluid cultures were negative for MRSA. Consequently, MRSA was definitively excluded as the etiology.

In most reported cases of severe pericarditis or cardiac tamponade associated with influenza virus infection, Influenza A is the causative subtype (37–39). Isolated pericarditis (without concomitant myocarditis) is frequently associated with pericardial effusions (40). Males and older patients are more likely to present with isolated pericarditis, whereas females and younger patients more commonly develop myopericarditis (40). Patients of any age presenting with chest pain, tachycardia, and hemodynamic instability occurring within 2–4 weeks of influenza-like illness onset should be evaluated for cardiac involvement (pericarditis or myopericarditis) (40). In this case, the patient presented with pericardial effusion but lacked other typical features such as recent influenza-like respiratory symptoms or chest pain, resulting in a low clinical suspicion for influenza-associated pericarditis.

Fungal pericarditis is rare. (1 → 3)-β-D-Glucan (BDG), a component of most fungal cell walls (except *Cryptococcus* and *Mucorales*), serves as a surrogate serum biomarker for diagnosing invasive fungal infections (IFIs) (41–43). BDG has been used in the diagnosis of IFIs, including *Pneumocystis jirovecii* pneumonia (PJP), cryptococcosis, and others, in people living with HIV (PLWH) (44–46). Several studies evaluating serum BDG (using the Fungitell assay with varying diagnostic cutoffs) for PJP diagnosis in PLWH demonstrated sensitivities of 90%–100% and specificities of 61.3–96.4% (44–46). However, for cryptococcosis diagnosis, BDG sensitivity in cerebrospinal fluid (CSF) is 89% (specificity 85%), whereas in serum, sensitivity is 79% (specificity 61%) (47). Currently, data on the sensitivity and specificity of serum BDG for diagnosing fungal pericarditis are lacking. In this case, the patient's serum BDG was negative. However, a fungal etiology could not be definitively excluded based on this result alone. Subsequently, culture of the pericardial fluid grew *Nocardia* species, with no fungal growth. The subsequent clinical response to *Nocardia*-directed antibiotic therapy, without antifungal agents, further supported *Nocardia* as the causative pathogen and and argued against a fungal etiology.

Microbiological identification remains the gold standard for diagnosing *Nocardia* pericarditis. However, in AIDS patients with negative initial pericardial fluid cultures and poor response to empirical therapy, extending the culture incubation period to at least 10 days is necessary to reduce the risk of false-negative results (16). Notably, the diagnostic approach (e.g., prolonged culture) and therapeutic resources (e.g., comprehensive susceptibility testing) employed in this case may be challenging to implement in resource-limited settings. In such settings, clinicians should prioritize basic microbiological tests (Gram stain and partial acid-fast stain) to facilitate the early suspicion/detection of *Nocardia* and consider initiating empirical therapy with a regimen of TMP-SMZ combined with amikacin, even without species identification. Additionally, pericardiocentesis for diagnostic sampling and therapeutic drainage should be emphasized as a critical intervention, irrespective of available diagnostic resources. As summarized in Table 2, the constellation of fever, dyspnea, chest pain, and pericardial effusion, particularly if accompanied by the isolation of slow-growing, branching Gram-positive bacilli exhibiting partial acid-fastness from pericardial fluid, should raise strong suspicion for *Nocardia* infection.

*Nocardia* infections are characterized by potential for rapid progression and a propensity for relapse or recrudescence (16). In this case, despite massive purulent pericardial effusion and rapid clinical deterioration, successful treatment relied on accurate microbiological identification, timely initiation of appropriate antibiotic therapy, and effective pericardiocentesis drainage.

Empirical antibiotic therapy should be initiated promptly when *Nocardia* infection is suspected. Trimethoprim-sulfamethoxazole (TMP-SMZ) is the established first-line agent for nocardiosis (48). Antimicrobial selection should consider factors including local epidemiology, clinical presentation (severity, sites of involvement), patient-specific factors (e.g., comorbidities, immunosuppression), antimicrobial susceptibility profiles, and potential adverse effects (2, 49–51). For isolates resistant to sulfonamides or patients intolerant of TMP-SMZ, alternatives include high-dose TMP-SMZ (if tolerated), combination therapy, or other active agents such as carbapenems (imipenem, meropenem) or linezolid (52, 53). Immunocompromised patients typically require extended antimicrobial therapy (minimum 6–12 months) (48). Early diagnosis, prompt initiation of therapy, adequate dosing, and adherence to the prolonged treatment duration are essential for cure and preventing relapse.

The favorable clinical and radiological response observed in our patient following the initiation of appropriate *Nocardia*-directed therapy (initially ceftriaxone/amikacin, transitioning to long-term TMP-SMZ -based combination) underscores its efficacy. This case highlights the necessity of including *Nocardia* pericarditis in the differential diagnosis of immunosuppressed patients presenting with purulent pericarditis. Early identification, accurate diagnosis, prompt initiation of appropriate treatment, and effective management are critical for improving patient outcomes. Crucial management components include ensuring timely pericardial puncture for diagnosis and drainage, administering an extended course of antimicrobial therapy, and closely monitoring for potential drug-related adverse effects.

The primary limitation is the inability to identify the *Nocardia* isolate to the species level due to resource constraints, specifically the lack of access to molecular techniques such as 16S rRNA sequencing. This is clinically significant due to interspecies variations in antimicrobial susceptibility (2, 54), which potentially compromise optimal therapy. Future studies and clinical practice should prioritize molecular identification for species-level diagnosis to enable species-directed therapy. Furthermore, as a single-center case report, the generalizability of our findings is limited. Multicenter studies with larger cohorts are needed to validate the proposed diagnostic and therapeutic approach.

*Nocardia* pericarditis is a rare but potentially fatal opportunistic infection that should be considered in the differential diagnosis of purulent pericarditis. Microbiological culture remains the diagnostic gold standard. Optimal outcomes require timely diagnosis, therapeutic pericardial drainage, and prolonged antimicrobial therapy.

## Data Availability

The datasets presented in this study can be found in online repositories. The names of the repository/repositories and accession number(s) can be found in the article/Supplementary Material.
